# Bilateral Iris Metastasis of Small Cell Lung Carcinoma: A Case Report and Systematic Review

**DOI:** 10.3390/clinpract16070118

**Published:** 2026-06-23

**Authors:** Stipe Vidović, Egon Biuk, Greta Biuk, Marija Jelić Vuković, Maja Vinković, Andrijana Kopić, Dubravka Biuk

**Affiliations:** 1 Clinic for Eye Diseases, Clinical Hospital Centre Osijek, 31000 Osijek, Croatia; 2 Faculty of Medicine Osijek, Josip Juraj Strossmayer University of Osijek, 31000 Osijek, Croatiagbiuk@mefos.hr (G.B.); 3 Department of Orthopedics, Clinical Hospital Centre Osijek, 31000 Osijek, Croatia

**Keywords:** small cell lung carcinoma, iris metastasis, bilateral iris metastasis, uveal metastasis, intraocular metastasis, ocular metastasis, secondary glaucoma, iris neovascularization, case report, systematic review

## Abstract

**Objective:** To report a rare case of bilateral iris metastasis from small cell lung carcinoma (SCLC) and systematically review the literature on SCLC-associated iris metastases, with emphasis on clinical presentation, management, and outcomes. **Materials and Methods:** A systematic literature review was conducted in accordance with the Preferred Reporting Items for Systematic Reviews and Meta-Analyses (PRISMA) guidelines. PubMed, ScienceDirect, Scopus, and Web of Science were comprehensively searched on 10 July 2025. Eligible studies included English-language reports of iris metastasis originating from SCLC in human subjects. **Case report:** A 58-year-old woman with previously treated SCLC developed bilateral iris metastases one year after complete remission of the primary tumor. Ophthalmic examination revealed whitish-gray, vascularized iris masses with iridocorneal angle involvement, associated with secondary angle-closure glaucoma and markedly elevated intraocular pressure (48 mm Hg) in the left eye. Cyclocryotherapy, preceded by systemic and topical antiglaucoma therapy, resulted in pain relief and a reduction in intraocular pressure; the patient died four months later due to pneumonia. **Results (Systematic Review):** Seventeen studies comprising 17 patients were included; the median age was 60 years, and 64.7% were male. The median interval from SCLC diagnosis to ocular presentation was 4 months, although iris metastasis was occasionally the initial or concurrent manifestation of disease. The most common presenting features were visual impairment (58.8%), ocular pain (41.2%), and elevated intraocular pressure (41.2%), while iris neovascularization (35.3%) and synechiae (29.4%) were also frequent. Bilateral involvement was reported in only one previous case. Treatment approaches were heterogeneous and included antiglaucoma therapy, systemic chemotherapy, local radiotherapy, anti-VEGF therapy, and enucleation. Among patients with available follow-up (n = 12), 58.3% died within a median follow-up of 7.5 months. **Conclusions:** Bilateral iris metastasis from SCLC is rare and may occur as a manifestation of recurrent disease after remission. It is an aggressive condition characterized by nonspecific ocular symptoms, variable management, and poor survival, underscoring the importance of early recognition and the need for evidence-based diagnostic and therapeutic strategies.

## 1. Introduction

Uveal metastasis is the most common form of intraocular malignancy in adults, occurring in approximately 2% to 9% of patients with advanced carcinomas [1,2]. The choroid accounts for the vast majority of cases, whereas metastases to the iris and ciliary body are distinctly uncommon [1,2]. This distribution likely reflects differences in vascular anatomy, as the iris is less richly vascularized than the posterior uveal tract [3]. Uveal metastases typically arise through hematogenous dissemination from distant primary tumors, most commonly breast carcinoma and lung carcinoma, followed by cutaneous melanoma and other solid malignancies [4].

Although iris metastases are rare overall, their clinical relevance is substantial because they may present with pain, blurred vision, secondary glaucoma, iris neovascularization, or other anterior segment findings that can mimic inflammatory or primary ocular disorders [5]. In the largest retrospective series to date, Shields et al. analyzed 104 cases of iris metastasis from systemic malignancies and found that breast cancer was the most frequent primary tumor (33%), followed by lung cancer (27%) and cutaneous melanoma (12%) [5]. However, evidence specifically addressing small cell lung carcinoma (SCLC) remains scarce.

SCLC is an aggressive malignancy characterized by rapid growth, early dissemination, and poor overall prognosis [6]. Ocular metastases from SCLC have been reported, yet detailed evidence regarding iris involvement, particularly with respect to clinical presentation, management, and outcomes, remains restricted to isolated case reports [5]. Bilateral iris metastasis appears to be exceptionally rare in this setting [7]. Consequently, disease-specific evidence to inform the recognition and management of this rare ophthalmic presentation remains limited.

Given the rarity of this presentation and the absence of a focused synthesis of the published literature, the present study has two aims: first to report a rare case of bilateral iris metastasis from SCLC; and second, to systematically review previously published cases of SCLC-associated iris metastasis, with emphasis on patient demographics, ocular symptoms and signs, diagnostic methods, therapeutic approaches, and clinical outcomes.

## 2. Case Presentation

A 58-year-old woman was diagnosed with small-cell lung carcinoma (SCLC) of the right lung in May 2017. She underwent six cycles of chemotherapy with cisplatin and gemcitabine, with dexamethasone as premedication, administered every three weeks. After the fourth cycle, a control chest X-ray showed marked regression of the disease. Following the sixth cycle, imaging confirmed complete remission of the primary tumor.

One year later, the patient presented with neurological symptoms, and brain metastases were confirmed via computed tomography (CT). Two weeks thereafter, she reported severe pain in her left eye and was referred to an ophthalmologist.

On examination, best corrected visual acuity (BCVA) was 1.0 in the right eye and no light perception (NLP) in the left eye. Medical history revealed longstanding poor vision in the left eye due to childhood ocular trauma. Intraocular pressure (IOP) was within normal limits in the right eye and measured 48 mm Hg in the left.

**Figure 1:**
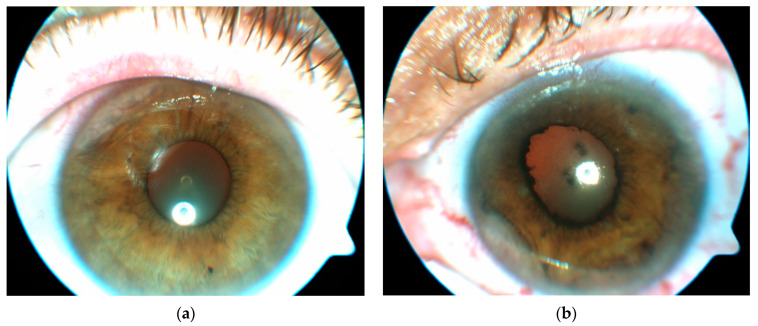
(**a**) A whitish-gray tumor mass at 10–12 o’clock (right eye); (**b**) A whitish-gray tumor mass at 1–8 o’clock (left eye).

Slit-lamp examination revealed whitish-gray, vascularized, solid masses arising from the iridocorneal angle. These lesions extended from 10 to 12 o’clock in the right eye and from 1 to 8 o’clock in the left eye, causing significant iris distortion (Figure 1). Gonioscopy confirmed these findings, particularly in the left eye, where the angle was closed by peripheral anterior synechiae. The optic disk appeared normal in the right eye, while optic atrophy was noted in the left.

The patient was admitted to the Department of Ophthalmology and initiated on anti-glaucoma therapy, including intravenous 20% mannitol (200 mL daily for two days, subsequently discontinued), oral acetazolamide 250 mg three times daily with potassium chloride supplementation, and topical therapy with timolol, brinzolamide, and bimatoprost administered twice daily.

B-scan ultrasonography showed no intraocular or retrobulbar masses. However, fluorescein iridography demonstrated vascularized lesions. Given the characteristic appearance, prior diagnosis of SCLC, and the patient’s poor general condition, a biopsy was not performed.

On the third day of hospitalization, cyclocryotherapy was performed in the left eye, resulting in a reduction of intraocular pressure (IOP) to 28 mmHg, after which oral acetazolamide was discontinued. At a two-week follow-up, IOP remained at 28 mmHg but without associated pain under continued topical therapy. Although IOP remained above the normal range, this was considered an acceptable outcome in the context of advanced metastatic disease, given the absence of pain and the primarily palliative treatment goal. Further aggressive interventions were not pursued due to the patient’s poor systemic condition and limited life expectancy.

The patient’s general health deteriorated rapidly, and she died of pneumonia four months after the ophthalmic intervention.

## 3. Materials and Methods

The following paragraphs describe the methodology of a systematic review on iris metastasis originating from small-cell lung cancer, conducted in accordance with the Preferred Reporting Items for Systematic Reviews and Meta-Analyses (PRISMA) guidelines [8]. The PRISMA 2020 Checklist is provided in the Supplementary Materials (Supplementary File S1). This systematic review was not registered in any review registry.

### 3.1. Eligibility Criteria

Eligibility criteria are summarized in Table 1.

### 3.2. Information Sources and Search Strategy

A literature search was performed on 10 July 2025 by two independent reviewers (S.V. and D.B.) using the following electronic databases: PubMed, ScienceDirect, Scopus, and Web of Science. Search strategies were developed using Boolean operators and adapted for each database (Table 2). Filters were applied where appropriate, including language (English), study type, and human subjects.

### 3.3. Selection Process, Data Collection, and Extracted Variables

After removing duplicate records, two reviewers (S.V. and D.B.) independently conducted a two-step screening process. First, the titles and abstracts of all retrieved articles were reviewed to identify potentially relevant studies. Articles that appeared to meet the inclusion criteria, or for which relevance could not be determined with certainty, were selected for full-text evaluation. The eligibility of each full-text article was then assessed using the predefined inclusion and exclusion criteria outlined in Table 1. Studies that failed to meet these criteria were excluded, and the reasons for exclusion were documented. To ensure comprehensive coverage of the literature, the reference lists of all included articles were also hand-searched (S.V. and D.B.) to identify any additional relevant studies not captured in the initial database search.

For each study that met the inclusion criteria, data extraction was performed independently by both reviewers (S.V. and D.B.) using a standardized data extraction form developed a priori. Any discrepancies during the selection or extraction process were resolved through discussion and consensus. If consensus could not be reached, a third reviewer (A.K.) was available to arbitrate, although this was not required.

The following variables were extracted when reported: author, year of publication, country, study design, sample size, age (years), sex (male/female), ocular-related symptoms and clinical findings, method of diagnosis, treatment modality, outcomes, and follow-up.

### 3.4. Study Risk of Bias Assessment

The methodological quality of the included studies was assessed using the Joanna Briggs Institute (JBI) Critical Appraisal Checklist for Case Reports [9]. Two independent reviewers (S.V. and D.B.) assessed each study using the relevant checklist, assigning one of the following ratings to each item: “Yes,” “No,” “Unclear,” or “Not applicable.” Any disagreements between the reviewers were resolved through consensus discussion. Each “Yes” response was awarded one point, while all other responses received zero points. The total score for each study was calculated as the sum of all “Yes” responses, and this score was expressed as a percentage of the maximum possible points. Studies were then categorized based on overall quality into one of three levels: low quality (<50%), moderate quality (50–74%), or high quality (≥75%).

### 3.5. Synthesis Methods

A narrative and descriptive synthesis was conducted. Categorical variables were summarized using frequencies and percentages based on the number of patients reporting each variable. Continuous variables were summarized using medians and interquartile ranges (IQRs). Studies with missing data for specific variables were excluded from those particular analyses but remained included in the overall synthesis. Results are presented in both descriptive and tabular formats. Given the descriptive nature of the review and the exclusive inclusion of single-patient case reports, no meta-analyses, quantitative effect measures, or formal certainty assessments were performed.

## 4. Results

### 4.1. Study Selection

A total of 518 records were identified through database searching. After removal of 35 duplicates, 483 records remained for screening.

Following title and abstract screening, 464 records were excluded based on predefined inclusion and exclusion criteria (Table 1). Nineteen articles were assessed for full-text eligibility, of which four were excluded for the following reasons: unspecified lung cancer subtype and insufficient clinical description of iris metastasis [10,11]; non–small cell lung carcinoma [12]; and incomplete data on treatment, outcomes, or follow-up [13].

**Figure 2:**
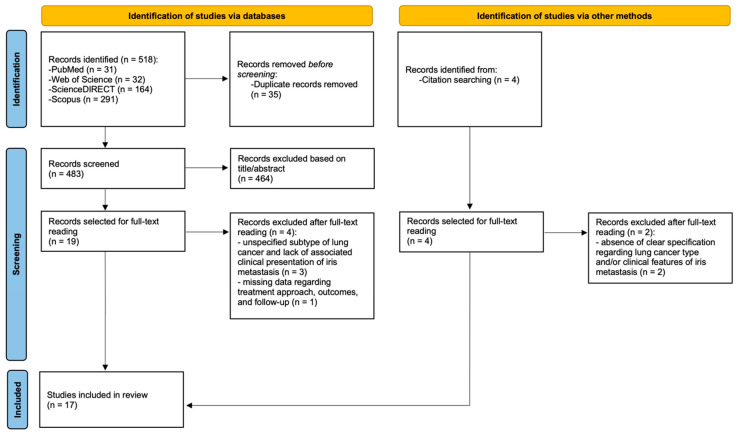
Literature search flow diagram.

Hand-searching of reference lists identified four additional studies. Two were excluded after full-text review due to unclear specification of the lung cancer subtype and/or insufficient clinical characterization of iris metastasis [5,14]. In total, 17 studies met the eligibility criteria and were included in the final analysis. The study selection process is illustrated in the PRISMA flow diagram (Figure 2).

### 4.2. Study Characteristics

A total of 17 case reports were included in the final analysis. The main study characteristics are summarized in Table 3.

### 4.3. Risk of Bias in Studies

Of the 17 included studies, 12 were classified as high quality, 4 as moderate quality, and 1 as low quality. Detailed quality assessment results are provided in the Supplementary File S2.

### 4.4. Summary of Included Studies

Seventeen patients with iris metastases from small-cell lung carcinoma (SCLC) were included. Eleven were male and six were female, with a median age of 60 years (IQR: 53–70).

Data on the interval between SCLC diagnosis and ophthalmic presentation were available for 13 patients, with a median interval of 4 months (IQR: 0.5–6). Most patients had received prior systemic therapy, including chemotherapy (n = 11), immunotherapy (n = 2), with 9 patients receiving combination therapy. In two cases, iris involvement was the initial manifestation leading to diagnosis of SCLC, while in one case, iris metastasis was identified concurrently with the primary tumor.

The most common ocular symptoms were blurred vision or visual impairment (10/17, 58.8%), followed by ocular pain (7/17, 41.2%) and ocular redness (3/17, 17.7%). Elevated intraocular pressure (IOP) was observed in 7 patients (41.2%), including 3 cases (17.7%) of neovascular glaucoma. Iris neovascularization was reported in 6 patients (35.3%), while synechiae were present in 5 patients (29.4%)—anterior in 3 and posterior in 2 cases. Bilateral iris metastases were identified in one patient.

Diagnostic confirmation of iris metastasis was obtained in 7 patients: histopathologically (n = 5), by aspiration biopsy (n = 1), and by anterior chamber fluid cytology (n = 1). Detailed patient characteristics and clinical findings are presented in Table 4.

With regard to treatment, antiglaucoma therapy was administered in patients with elevated IOP. Additional interventions included anti-VEGF therapy (n = 3), local ocular radiotherapy (n = 3), systemic chemotherapy (n = 7), and enucleation (n = 3).

Complete regression of iris metastases was achieved in 4 patients treated with systemic chemotherapy and/or local radiotherapy, while partial regression was observed in 6 patients. In 3 patients, no change in tumor size was observed.

Follow-up data were available for 12 patients, with a median duration of 7.5 months (IQR: 7.5–11). During follow-up, 7 patients died. Treatment modalities, outcomes, and follow-up data are summarized in Table 5.

## 5. Discussion

This study presents a rare case of bilateral iris metastasis from small cell lung carcinoma (SCLC), together with a systematic review of the available literature on this infrequent manifestation.

Regarding the time to ophthalmic clinical presentation after SCLC diagnosis, the median interval was 4 months, and most patients had already received therapy, including chemotherapy, radiotherapy, and/or immunotherapy. Notably, in three reported case reports, the initial pathology was detected in the iris, which subsequently led to the diagnosis of SCLC [17,21]. This suggests that suspicious iris lesions may warrant further systemic evaluation to rule out a primary site of metastasis, including SCLC.

Patients with iris metastases from SCLC commonly present with ocular symptoms such as eye pain, blurred vision, or visual impairment, as observed in our patient. Elevated intraocular pressure (IOP) was reported in nearly half of cases, and synechiae were present in approximately one-third of patients (Table 4). In our case, synechiae were observed in both eyes, with angle closure in the left eye. Iris neovascularization was also reported in about one-third of patients (Table 4). These findings are consistent with those reported by Shields et al. (2015), who analyzed 104 patients with iris metastases from various primary tumors and documented eye pain in 32% of cases, blurred vision in 30%, iris neovascularization in 27%, and secondary glaucoma in 37% [5]. In the same study, tumor color was most commonly yellow (66%), followed by brown (14%), red-orange (12%), and white or gray (7%), the latter corresponding to the appearance observed in our patient. Tumors were most frequently located in the peripheral or root region of the iris (69%), and most patients (78%) had a single lesion per eye [5]. Bilateral involvement was exceptionally rare. In our review, only one additional case of bilateral iris metastasis from SCLC was identified [7].

Differential diagnosis of iris metastases is broad and encompasses both neoplastic and non-neoplastic conditions. Primary iris melanoma, the most common primary malignant tumor of the iris, represents an important diagnostic consideration [31,32]. Other entities that may mimic iris metastases include iris nevus, iridociliary cysts, melanocytoma, leiomyoma, juvenile xanthogranuloma, lymphoma, granulomatous inflammatory lesions, iris vascular tumors, and metastases originating from other systemic malignancies [32,33]. Clinically, metastatic iris lesions are more associated with rapid progression, diffuse or multifocal involvement, prominent vascularization, ocular pain, inflammatory signs, and secondary elevation of intraocular pressure, whereas primary iris melanoma typically follows a more indolent clinical course [32,33]. Previous studies have identified breast and lung carcinomas as among the most common primary tumors associated with iris metastases, while prognosis is generally determined by systemic tumor burden rather than local ocular disease [5,34]. Furthermore, although a known history of systemic malignancy may strongly suggest metastatic disease, iris involvement may occasionally represent the initial manifestation leading to the diagnosis of SCLC, as observed in several cases included in our review [16,21].

Comprehensive ophthalmologic evaluation is essential for accurate diagnosis and treatment planning in patients with suspected iris metastases. Slit-lamp biomicroscopy and gonioscopy remain central to anterior segment assessment, while adjunctive imaging modalities, particularly ultrasound biomicroscopy (UBM) and anterior segment optical coherence tomography (AS-OCT), may further improve lesion characterization by assessing tumor extension, stromal infiltration, and angle involvement [33,35]. Although histopathological confirmation by iris biopsy remains the diagnostic gold standard, diagnosis is frequently established based on clinical findings and multimodal imaging, particularly in patients with disseminated malignancy, poor general condition, or when biopsy results are unlikely to alter therapeutic management. Consequently, several cases included in our review, including the present case, should be regarded as clinically presumed rather than histopathologically confirmed iris metastases [16,17,18,20,22,25,26,29,30]. Recent literature has further emphasized this diagnostic uncertainty by questioning whether all iris masses observed in patients with lung carcinoma necessarily represent true metastatic lesions, thereby underscoring the importance of careful differential diagnosis and histopathological confirmation whenever feasible [36].

Our patient received only symptomatic treatment aimed at lowering intraocular pressure (IOP). Similarly, a substantial proportion of patients included in this review did not receive specific ocular therapy beyond symptomatic management [7,15,16,17,18,19,22,23,26]. Among treated patients, the most commonly employed approaches were systemic platinum-based chemotherapy, local radiotherapy, and anti-VEGF therapy. Favorable ocular responses, including partial or complete regression of iris metastases, were observed following cisplatin/etoposide-based chemotherapy [22,23,26], orbital region radiotherapy [20], or intravitreal anti-VEGF treatment, particularly in cases with iris neovascularization and secondary glaucoma [15,18,25]. Conversely, less favorable outcomes were also reported despite similar therapeutic approaches, particularly in patients with widespread progressive systemic disease and limited response to systemic therapy [7,19,20,29]. These findings should therefore be interpreted cautiously, as the available evidence consists exclusively of single-patient case reports with substantial heterogeneity in treatment strategies, disease burden, follow-up duration, and outcome reporting. Consequently, no comparative conclusions regarding optimal management or treatment efficacy can be established. Nevertheless, the studies included in this systematic review suggest that, in clinical practice, management remains highly individualized and is frequently guided by systemic disease status, ocular pain, visual potential, intraocular pressure control, and overall life expectancy.

Regarding mortality, our patient died within four months after the diagnosis of iris metastasis due to clinical deterioration associated with pneumonia. Overall prognosis appeared poor. Follow-up data were available for 12 patients, with a median follow-up of 7.5 months (IQR: 7.5–11). Among these patients, 7 deaths were reported during follow-up, predominantly due to progressive disseminated SCLC, with follow-up durations before death ranging from 2 to 10 months after diagnosis of iris metastasis [7,15,19,23,24,25,26]. In the remaining five cases with available follow-up data, no mortality was reported during follow-up [17,21,27,28,30]. These findings are consistent with previous reports demonstrating poor survival among patients with iris metastases. In a retrospective study by Shields et al., including 85 patients with iris metastases from various primary tumors, the mortality rate was 87% over a median follow-up of 10 months [5], underscoring the aggressive nature of metastatic disease.

### Limitations and Further Directions

This study has several limitations. First, the small sample size, comprising 17 case reports and 17 patients, limits the robustness and generalizability of the findings. The inclusion of only case reports introduces an inherent risk of selection bias, and publication bias cannot be excluded, as unusual or clinically significant cases are more likely to be reported. Additionally, variability in the terminology used to describe ocular metastatic disease may have resulted in the omission of isolated relevant reports despite comprehensive database searching and manual reference screening.

Second, variability in clinical presentation, diagnostic approaches, treatment strategies, and reporting standards across studies limited comparability and precluded quantitative synthesis. Additionally, incomplete reporting of key clinical variables further constrained data analysis. Additionally, incomplete reporting of key clinical variables further constrained data analysis. In some cases, including our own, the diagnosis of iris metastasis was based on clinical and imaging findings without histopathological confirmation by iris biopsy, limiting diagnostic certainty and leaving the diagnosis presumptive rather than definitive.

Third, all included studies were retrospective in nature, and the absence of a meta-analysis limited the ability to perform a quantitative assessment. Consequently, interpretation of the findings was primarily descriptive. In addition, the descriptive design of the review and the exclusive inclusion of case reports precluded quantitative synthesis, formal certainty assessment, and comparative evaluation of treatment efficacy. The overall certainty of evidence is low, given that all included studies were case reports. These limitations reflect methodological challenges commonly encountered in systematic reviews of rare ocular malignancy-related conditions within ophthalmic oncology, where evidence synthesis is frequently constrained by limited patient numbers, retrospective study designs, and heterogeneous clinical reporting. Consequently, the findings of the present review should be interpreted cautiously and primarily as descriptive observations rather than definitive evidence for comparative diagnostic or therapeutic recommendations [37]. Furthermore, although methodological quality was assessed using the JBI Critical Appraisal Checklist, these ratings primarily reflect reporting completeness and methodological transparency rather than strength of evidence, causal inference, or comparative therapeutic efficacy [9]. Consequently, even well-documented case reports remain inherently low-level evidence and are particularly susceptible to publication bias, selective reporting, and uncontrolled confounding. Additionally, formal assessment of reporting bias was not performed due to the limited number and heterogeneity of included studies. Moreover, the focus on iris metastases secondary to SCLC, while clinically relevant, limits broader applicability. Comparative studies including other primary malignancies—such as breast cancer, non–small-cell lung cancer, or melanoma—may help identify disease-specific patterns.

Future research should prioritize larger, multicenter studies with standardized data collection and reporting. Prospective observational studies and collaborative registries may improve data quality and enable more robust analyses. Such efforts are essential for developing evidence-based diagnostic and therapeutic strategies for this rare condition.

## 6. Conclusions

This report describes a rare case of bilateral iris metastasis from small cell lung carcinoma occurring as a recurrence after complete remission, with ocular involvement representing an early manifestation of systemic disease progression. Consistent with our systematic review, this condition is characterized by nonspecific ocular symptoms, variable management, and poor survival, highlighting the need for early recognition and coordinated multicenter efforts to establish evidence-based diagnostic and therapeutic strategies.

## Figures and Tables

**Table 1:** Inclusion and exclusion criteria.

	Inclusion Criteria	Exclusion Criteria
Period of the study	All available literature up to the date of search	/
Language	English	Non-English languages
Study design	Case report, retrospective study (case–control studies or case series), prospective cohort study, cross-sectional study, meta-analysis, and systematic review	Conference abstracts, letters to the editor, commentaries, expert opinions, narrative reviews, personal communications, discussion, and editorials
Study population	Human subjects	Non-human studies
Primary tumor type	Small-cell lung carcinoma	All other tumor types
Metastasis site	Presence of iris metastasis	Presence of non-iris metastasis

**Table 2:** Information sources and Boolean operators used in systematic literature search.

Information Source (Electronic Database)	Boolean Operator
PubMed	((iris) AND (small-cell lung carcinoma)) AND (metastasis).
Web of Science	TS = (((iris) AND (small-cell lung carcinoma)) AND (metastasis)) Filters applied: Document Types (Article or Case Report), and Language (English).
ScienceDirect	((iris) AND (small-cell lung carcinoma)) AND (metastasis) Filters applied: Article type (review articles, research articles, and case reports), Language (English), and Access type (Open Access).
Scopus	((iris) AND (small-cell lung carcinoma)) AND (metastasis) AND (LIMIT-TO (SUBJAREA, “MEDI”)) AND (LIMIT-TO (DOCTYPE, “ar”)) AND (LIMIT-TO (EXACTKEYWORD, “Human”)) AND (LIMIT-TO (LANGUAGE, “English”)).

**Table 3:** Key characteristic of included studies.

Author	Year of Publication	Country	Study Design	Sample Size
Goto et al. [15]	2025	Japan	Case report	1
Ciftci et al. [7]	2024	Turkey	Case report	1
Huang and Zhang [16]	2023	China	Case report	1
Nguyen et al. [17]	2021	USA	Case report	1
Hidaka et al. [18]	2018	Japan	Case report	1
Chen et al. [19]	2018	Taiwan	Case report	1
Sakellakis et al. [20]	2016	Greece	Case report	1
Liu et al. [21]	2016	China	Case report	1
Hata and Inoue [22]	2014	Japan	Case report	1
Nikratowicz [23]	2013	Poland	Case report	1
Fukui et al. [24]	2012	Japan	Case report	1
Nakashima et al. [25]	2010	Japan	Case report	1
Alacacioğlu et al. [26]	2008	Turkey	Case report	1
Roenhorst [27]	2007	Netherland	Case report	1
Moura et al. [28]	2006	Canada	Case report	1
Ampil et al. [29]	1990	USA	Case report	1
Sierocki et al. [30]	1980	USA	Case report	1

**Table 4:** Key characteristics and clinical findings of patients with SCLC iris metastasis.

	Age (Years)	Sex (M/F)	Time to Ophthalmic Clinical Presentation After SCLC Diagnosis	Ocular-Related Symptoms and Clinical Findings
Goto et al. [15]	65	M	4 months (5 cycles of chemotherapy)	1 month history of ocular pain and blurred vision (OD) Visual acuity = 0.1 OD (uncorrectable) and 0.4 (corrected) OS IOP = 55 mm Hg (OD) (neovascular glaucoma), 11 mm Hg (OS) Corneal stromal edema (OD) Anterior chamber contained 3+ cells and iris neovascularization at the pupillary border (OD) Gonioscopy: whitish irregular mass from the anterior iris surface to the anterior part of the trabecular meshwork (OD) Method of SCLC iris metastasis diagnosis: anterior chamber fluid cytology (OD)
Ciftci et al. [7]	46	M	6 months (9 cycles of chemotherapy, immunotherapy, and cranial radiation)	1 week history of photophobia Visual acuity = 0.1 (corrected) OU Bilateral multiple vascularized iris masses with nodular configuration IOP = 12 mm Hg (OU) Method of SCLC iris metastasis diagnosis: aspiration biopsy
Huang and Zhang [16]	57	F	10 months (4 cycles of chemotherapy and 2 cycles of immunotherapy)	Right eye pain and blindness Positive relative afferent pupillary defect (RAPD) (OD). Conjunctival hyperemia and a large temporal gray irregular iris mass, peripheral anterior synechiae of the iris, and iris neovascularization
Nguyen et al. [17]	70	M	Initial pathology detected on the iris, directing towards a diagnosis of SCLC	3 week history of right eye redness and blurry vision Corrected visual acuity was 20/50 (OD) and 20/70 (OS) A collection of white, solid, fluffy material in anterior chamber (pseudohypopyon); inferiorly located (OD) Inferior iris infiltration and thickening with engorged adjacent stromal vessels (OD) Posterior synechiae formation (180° and 210°) (OD) Hyperreflective endothelial lesion (anterior OCT) (OD)
Hidaka et al. [18]	79	M	11 months (3 cycles of chemotherapy)	5-day history of right ocular pain and blurred vision Visual acuity = 0.1 OD (corrected) and 0.5 (corrected) OS IOP = 30 mm Hg (OD) (neovascular glaucoma); 10 mm Hg (OS) Positive relative afferent pupillary defect (RAPD) (OD) Conjunctival injection and multiple temporal gray irregular iris masses associated with peripheral anterior synechia (PAS) and neovascularization of the iris (OD) Iris masses OD (anterior OCT and UBM) and iris neovascularization (FAG)
Chen et al. [19]	60	M	NA	1 month history of redness and tearing of right eye Best corrected visual acuity was 0.5 OD and 0.6 OS IOP = 16 mmHg Grayish-white iris mass located between 8 o’clock and 10 o’clock pupillary margins (OD) (slit lamp biomicroscopy) Spectral-domain optical coherence tomography at presentation, showing thickening of the retina and inner retinal replacement by tumor (OS) Method of SCLC iris metastasis diagnosis: histopathology
Sakellakis et al. [20]	76	F	NA (6 cycles of chemotherapy and cranial radiation)	Visual impairment Secondary glaucoma OS (IOP not reported)
Liu et al. [21]	59	M	Initial pathology detected on the iris, directing towards a diagnosis of SCLC	6-month history of right eye pain and redness Corrected visual acuity was 20/30 (OD) and 20/40 (OS) IOP = 37 mm Hg (OD) (neovascular glaucoma), 15 mm Hg (OS) Vascularized iris mass at 10 o’clock to 1 o’clock (OD) (UBM and orbital MRI) Method of SCLC iris metastasis diagnosis: histopathology
Hata and Inoue [22]	54	M	4 months (chemotherapy, chemoradiotherapy, and radiotherapy)	Blurred vision (OD) Corrected visual acuity was 0.9 (OD) Supertemporal 5 mm iris mass (OD)
Nikratowicz [23]	43	F	6 months (5 cycles of chemotherapy and chest radiation)	Redness and slightly decreased vision (OS) Decreased best corrected visual acuity (BCVA) to 0.9 (OS) IOP = 22 mm Hg (OS)
Fukui et al. [24]	70	M	6 months (4 cycles of chemotherapy)	Blurred vision due to detachment of retina (OS) Incidental finding of pale yellow iris mass at 1 o’clock meridian (OS) IOP = 13 mm Hg (OD), 12 mm Hg (OS) Method of SCLC iris metastasis diagnosis: histopathology
Nakashima et al. [25]	52	M	14 months (7 cycles of chemotherapy and radiotherapy)	1 month history of blurred vision (OD) IOP = 19 mm Hg (OD) and 15 mm Hg (OS) Inferonasal iris mass, associated with peripheral anterior synechia and neovascularization of the iris (OD) Rise in IOP to 36 mm Hg (OD) over a 6-week period
Alacacioğlu et al. [26]	50	M	Iris metastasis identified at the time of SCLC diagnosis	2-day history of right eye pain Vegetative lesion on iris (slit lamp biomicroscopy)
Roenhorst [27]	58	F	NA (5 cycles of chemotherapy and radiotherapy)	2 week history of white spot in her right eye 2 amelanotic tumors in iris; 4 o’clock and 6 o’clock meridian (OD) Method of SCLC iris metastasis diagnosis: histopathology
Moura et al. [28]	69	F	6 months (chemotherapy and radiotherapy)	1 month history of left eye pain Multiple grayish-white nodules (anterior iris surface), segmented atrophy of the iris, rubeosis iridis, and posterior synechiae almost causing pupillary block IOP = 40 mm Hg (OS) Method of SCLC iris metastasis diagnosis: histopathology
Ampil et al. [29]	61	M	NA (chemotherapy and radiotherapy)	Blurring vision, headache, and conjunctivitis Tumors in iris; 2 o’clock meridian (OS) (slit lamp biomicroscopy)
Sierocki et al. [30]	71	M	NA	Blurring vision IOP = 32 mmHg (OD) and 20 mm Hg (OS) Solid-appearing, pinkish-white, nodular mass on the iris temporally (OD)

M = Male; F = Female; SCLC = Small-cell lung carcinoma; IOP = Intraocular pressure; OD = Oculi dextri; OS = Oculi sinistri; OU = oculus uterque; OCT = optical coherence tomography; FAG = fluorescein angiography; UBM = ultrasound biomicroscopy; MRI = Magnetic resonance imaging.

**Table 5:** Treatment modality, outcomes, and follow-up of patients with SCLC iris metastasis.

	Treatment Modality	Outcomes	Follow-Up
Goto et al. [15]	Ophthalmic treatment: topical latanoprost, brinzolamide, timolol maleate, oral acetazolamide, intravenous hypertonic mannitol, and intravitreal aflibercept	Five days after the aflibercept injection, the iris tumor and neovascularization decreased in size; IOP was 18 mm Hg (OD); ocular pain and nausea resolved. Four months later, the patient succumbed to multiorgan SCLC metastasis.	4 months
Ciftci et al. [7]	Ophthalmic treatment: none	No regression of primary or metastatic foci after chemotherapy. Two months after detection of iris metastasis, the patient died due to respiratory failure.	2 months
Huang and Zhang [16]	Ophthalmic treatment: none Systemic treatment: carboplatin/etoposide + atezolizumab (4 cycles), followed by atezolizumab maintenance (2 cycles); after progression and diagnosis of iris metastasis: irinotecan + atezolizumab + anlotinib (1 cycle)	Visual acuity improved with significant pain relief in the right eye. Iris metastasis markedly decreased.	NA
Nguyen et al. [17]	Ophthalmic treatment: None Systemic treatment: etoposide and carboplatin (1 cycle)	Significant regression of pseudohypopyon after two months of chemotherapy, with near-complete resolution of the endothelial lesion.	2 months
Hidaka et al. [18]	Ophthalmic treatment: topical latanoprost, brinzolamide, timolol maleate, and oral acetazolamide (500 mg/day). Due to persistent elevated IOP (33 mm Hg), intravitreal bevacizumab (1.25 mg) was administered one month later.	Rapid regression of iris masses and resolution of neovascularization after bevacizumab injection. Trabeculectomy was performed due to uncontrolled IOP (OD). After trabeculectomy, his right IOP decreased to 10 mm Hg. Nevertheless, the right optic disk had atrophied (light perception positive; OD)	NA
Chen et al. [19]	Ophthalmic treatment: none Systemic treatment: chemotherapy (regimen and number of cycles not reported) and brain radiotherapy	Two months after treatment, multiple iris nodules reappeared (OD); retinal lesion (OS) progressed. Approximately eight months after diagnosis, the patient died due to disseminated SCLC.	8 months
Sakellakis et al. [20]	Ophthalmic treatment: none Systemic treatment: carboplatin (area under the curve, AUC 6) + etoposide 120 mg/m^2^ every 21 days (6 cycles), followed by cranial radiotherapy (30 Gy); after progression: pazopanib 800 mg daily, then topotecan 2.3 mg/m^2^ on days 1–5 every 21 days	Disease progression after three months; therapy switched to topotecan. Further progression after an additional three months; palliative care initiated due to clinical deterioration.	NA
Liu et al. [21]	Ophthalmic treatment: Despite 14 days of therapy (carteolol hydrochloride, brimonidine tartrate, oral methazolamide and 20% mannitol intravenous injection) IOP continued to be poorly controlled. Enucleation (OD) Systemic treatment: cisplatin 80 mg/m^2^ (day 1) + etoposide 100 mg/m^2^ (days 1–3) every 21 days (1 cycle)	Leukopenia developed after one cycle of chemotherapy; further treatment was discontinued.	12 months
Hata and Inoue [22]	Ophthalmic treatment: none Systemic treatment: cisplatin + etoposide (dose and number of cycles not reported); due to regrowth of iris metastasis, orbital radiotherapy with 7-MeV electrons was administered (40 Gy in 20 fractions; 2 Gy/day, 5 days/week)	Rapid tumor regression after initiation of radiotherapy, with complete resolution within 30 days. Mild radiation keratitis, conjunctivitis, and dermatitis resolved after treatment.	NA
Nikratowicz [23]	Ophthalmic treatment: none Systemic treatment: cisplatin + etoposide (5 cycles) with chest radiotherapy (58 Gy); after development of iris metastasis: re-chemotherapy with cisplatin + etoposide (7 cycles)	One month after treatment: regression of iris tumor. Three months after treatment: complete regression of metastatic nodules with improved BCVA. The patient died due to disseminated SCLC one year after initial diagnosis.	7 months
Fukui et al. [24]	Ophthalmic treatment: none	The iris tumor increased in size Ten months after initial SCLC diagnosis, the patient died of respiratory failure.	10 months
Nakashima et al. [25]	Ophthalmic treatment: IOP (OD) poorly controlled despite timolol maleate, brinzolamide, latanoprost, and oral acetazolamide. bevacizumab (single injection 1.25 mg)	Two weeks after bevacizumab injection: regression of iris tumor, resolution of neovascularization, and IOP reduced to 18 mm Hg. After additional chemotherapy: iris atrophy without recurrence. The patient died eight months later due to systemic SCLC metastasis.	9 months
Alacacioğlu et al. [26]	Ophthalmic treatment: none Systemic treatment: cisplatin + etoposide chemotherapy (5 cycles); cranial radiotherapy for neurohypophysial and infundibular metastases. Iris metastasis regressed after the second chemotherapy cycle	Iris lesion resolved after the second chemotherapy cycle. The patient died due to nosocomial pneumonia after five cycles (6 months).	6 months
Roenhorst [27]	Ophthalmic treatment: external beam radiotherapy (25 × 1.8 Gy; total dose 45 Gy); regression observed after 25.2 Gy and further reduction after 41.4 Gy, with pain relief; secondary glaucoma developed during treatment	The iris tumor decreased in size, and the pain vanished As a result of radiotherapy, secondary glaucoma developed (resistant to local therapy) Enucleation of the right eye was performed 18 months after radiotherapy.	60 months
Moura et al. [28]	Ophthalmic treatment: radiotherapy	After two months, the mass became atrophic and fibrotic; IOP decreased to 22 mm Hg with therapy. Recurrence after three months; multiple iris nodules developed. Enucleation performed.	22 months
Ampil et al. [29]	Ophthalmic treatment: radiotherapy	Neoplastic condition rapidly progressed, and the patient died	NA
Sierocki et al. [30]	Ophthalmic treatment: NA Systemic treatment: cisplatin + etoposide for progressive mediastinal and axillary disease; corticosteroids and radiotherapy for superior vena cava obstruction; after iris metastasis: vindesine 3 mg/m^2^ intravenously every 7 days, resulting in complete regression of the iris lesion after 4 weeks	Complete regression of the iris tumor four weeks after chemotherapy initiation.	7 months

SCLC = Small-cell lung carcinoma; IOP = Intraocular pressure; OD = Oculi dextri; OS = Oculi sinistri; OU = oculus uterque; Gy = Gray; AUC = area under the curve.

## Data Availability

The data supporting the findings of the literature review are available from the corresponding authors upon reasonable request, in accordance with ethical standards and data privacy regulations.

## Supplementary Materials

The following supporting information can be downloaded at: https://www.mdpi.com/article/10.3390/clinpract16070118/s1, File S1: The PRISMA 2020 Checklist; File S2: Methodological quality of included studies.
