# Supplementary material for: Bilateral Iris Metastasis of Small Cell Lung Carcinoma: A Case Report and Systematic Review
Source: Clin Pract. 2026 Jun 23;16(7):118. doi: 10.3390/clinpract16070118 (PMC13407523; doi:10.3390/clinpract16070118)
Supplement: Supplementary file 1 [file clinpract-16-00118-s001.zip › Supplementary File S2.pdf]

Table. Methodological quality of included studies according to the Joanna Briggs Institute (JBI) Critical Appraisal Checklist for Case Reports.

	**Methodological quality (JBI Critical Appraisal Checklist for Case report)**								
	Q1	Q2	Q3	Q4	Q5	Q6	Q7	Q8	Overall score
Goto et al. [15]	Yes	Yes	Yes	Yes	Yes	Yes	Yes	Yes	100% (High quality)
Ciftci et al. [7]	Yes	Yes	Yes	Yes	Yes	Yes	Yes	Yes	100% (High quality)
Huang and Zhang [16]	Yes	Yes	No	Yes	Yes	No	NA	No	62.5% (Moderate quality)
Nguyen et al. [17]	Yes	Yes	Yes	Yes	Yes	No	NA	Yes	75% (High quality)
Hidaka et al. [18]	Yes	Yes	Yes	Yes	Yes	No	No	Yes	62.5% (Moderate quality)
Chen et al. [19]	Yes	Yes	Yes	Yes	Yes	Yes	Yes	Yes	100% (High quality)
Sakellakis et al. [20]	Yes	Yes	No	No	Yes	No	No	No	37.5% (Low quality)
Liu et al. [21]	Yes	Yes	Yes	Yes	Yes	Yes	Yes	Yes	100% (High quality)
Hata and Inoue [22]	Yes	Yes	No	Yes	Yes	No	Yes	Yes	75% (High quality)
Nikratowicz [23]	Yes	Yes	Yes	Yes	No	Yes	Yes	Yes	87.5% (High quality)
Fukui et al. [24]	Yes	Yes	Yes	Yes	Yes	Yes	Yes	Yes	100% (High quality)
Nakashima et al. [25]	Yes	Yes	Yes	Yes	Yes	Yes	Yes	Yes	100% (High quality)
Alacacioğlu et al. [26]	Yes	Yes	No	Yes	Yes	Yes	Yes	Yes	87.5% (High quality)
Roenhorst [27]	Yes	Yes	No	No	Yes	Yes	Yes	Yes	75% (High quality)
Moura et al. [28]	Yes	Yes	Yes	Yes	Yes	Yes	Yes	Yes	100% (High quality)
Ampil et al. [29]	Yes	Yes	No	No	Yes	No	No	Yes	50% (Moderate quality)
Sierocki et al. [30]	Yes	Yes	Yes	Yes	Yes	Yes	Yes	Yes	100% (High quality)

Q1 (question 1) = Were patient's demographic characteristics clearly described?
Q2 (question 2) = Was the patient's history clearly described and presented as a timeline?
Q3 (question 3) = Was the current clinical condition of the patient on presentation clearly described?
Q4 (question 4) = Were diagnostic tests or assessment methods and the results clearly described?
Q5 (question 5) = Was the intervention(s) or treatment procedure(s) clearly described?
Q6 (question 6) = Was the post-intervention clinical condition clearly described?
Q7 (question 7) = Were adverse events (harms) or unanticipated events identified and described?
Q8 (question 8) = Does the case report provide takeaway lessons?
U = Unclear, NA = Not applicable.
